# Biomechanical Modeling of the Forces Applied to Closed Incisions During Single-Use Negative Pressure Wound Therapy

**Published:** 2016-07-13

**Authors:** John Loveluck, Tom Copeland, Jason Hill, Allan Hunt, Robin Martin

**Affiliations:** ^a^42 Technology Ltd, St Ives, Cambridgeshire, United Kingdom; ^b^Dynamiq Engineering Ltd, Rugeley, Staffordshire, United Kingdom; ^c^Advanced Wound Management, Smith & Nephew Ltd, Hull, United Kingdom

**Keywords:** negative pressure wound therapy (NPWT), closed incisions, surgical site complications, FEA, incisional NPWT

## Abstract

**Objectives:** The use of negative pressure wound therapy (NPWT) on closed surgical incisions is an emerging technology that may reduce the incidence of complications such as surgical site infections. One of the mechanisms through which incisional NPWT is thought to operate is the reduction of lateral tension across the wound. **Methods:** Finite element analysis computer modeling and biomechanical testing with Syndaver SynTissue™ synthetic skin were used to explore the biomechanical forces in the presence of the PICO^⋄^ (Smith & Nephew Ltd, Hull, United Kingdom) negative pressure wound therapy system on a sutured incision. **Results:** Finite element analysis modeling showed that the force on an individual suture reduced to 43% of the force without negative pressure (from 1.31 to 0.56 N) at −40 mm Hg and to 31% (from 1.31 to 0.40 N) at −80 mm Hg. Biomechanical testing showed that at a pressure of −80 mm Hg, 55% more force is required for deformations in the tissue compared with the situation where no negative pressure wound therapy dressing is active. The force required for the same deformation at −120 mm Hg is only 10% greater than at −80 mm Hg, suggesting that most of the effect is achieved at −80 mm Hg. **Conclusions:** The results show that a canister-less single-use NPWT device is able to reduce the lateral tension across a closed incision, which may explain observed clinical reductions in surgical site complications with incisional NPWT.

After a long history of use in all manner of open wounds, negative pressure wound therapy (NPWT) is finding increasing use as a prophylactic application on closed incisions to reduce the incidence of surgical site complications.^1^^-^4 In recent clinical trials, a new generation of single-use disposable NPWT devices has also been shown to reduce surgical site complications.^5^^-^8 This is significant, as lower-cost single-use devices could make it economically viable to consider the so-called “incisional NPWT” as a preventative measure in higher-risk closed surgical incisions on a much wider scale than with traditional NPWT devices. 9 Experimental and clinical investigations have illuminated some of the mechanisms of these effects. Reductions in tissue edema, seroma, and hematoma and increases in wound breaking strengths have been reported.^10^^-^13 An additional mechanism through which incisional NPWT may exert its effects is through a compressive force that reduces the tension inherent in the sutures or staples. One study has used finite element analysis (FEA) and in vitro skin models to show that application of negative pressure to an open-cell foam-based single-use NPWT system can normalize forces around surgical incisions.^14^ Reduction of lateral force may be important in resisting mechanical stresses that retard closure and predispose incisional wounds to dehiscence and infection.^13^ Minimizing lateral tension may also contribute to a reduction in scar formation.^15^^,^^16^ The purpose of the present study was to assess the biomechanical forces applied to closed incisions from a single-use NPWT device (PICO; Smith & Nephew Ltd, Hull, United Kingdom) that uses a canister-less multilayer dressing technology to distribute NPWT, instead of traditional polyurethane “black” NPWT foam.^17^^,^^18^

## METHODS

### FEA computer model

**Figure 1:**
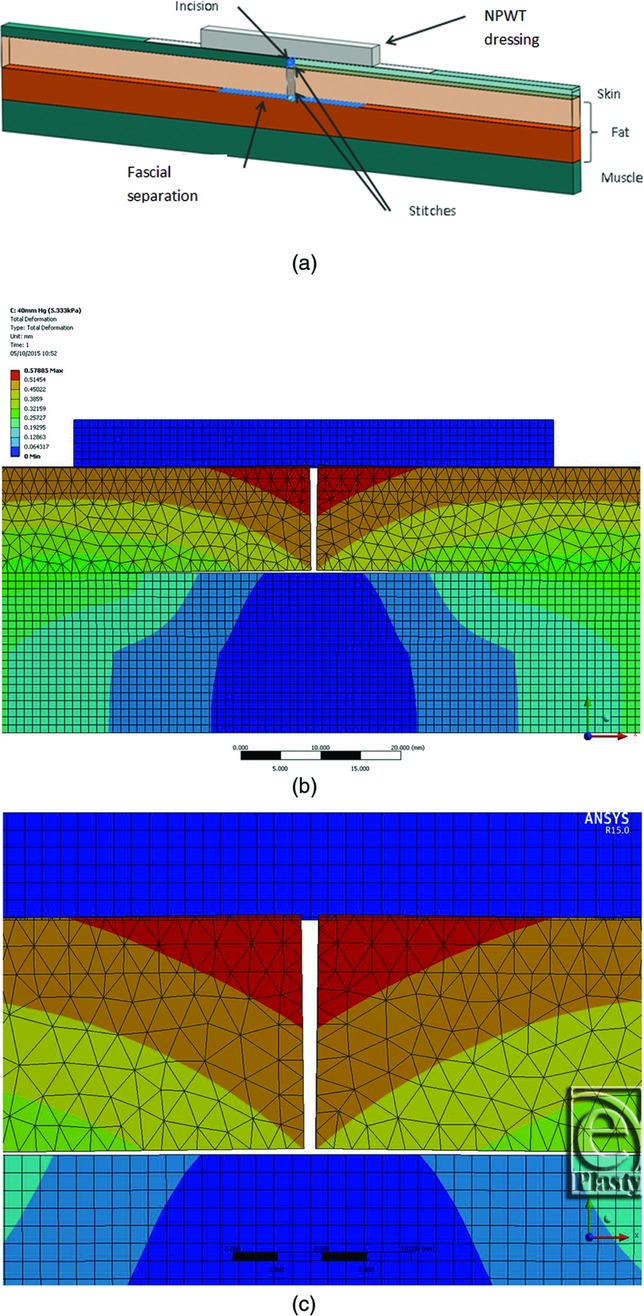
Three-dimensional finite element analysis computer model of skin incision. (a) The model comprises 3 layers: skin, fat, and muscle. The incision consists of a vertical cut through the skin and 10 mm into the fat. A 50-mm wide horizontal fascial separation was created at the bottom of the incision. (b) Pattern of forces around skin incision in millimeters of tissue deformation from its starting position due to the inherent lateral tensions in the tissue when the incision is made (blue is small; red is larger) The plane of the section is across the incision. (c) Close-up from (b).

The model was created using ANSYS FEA software (version 14.0; ANSYS UK Ltd, Horsham, United Kingdom). The incision geometry is shown in Figure 1*a*. The human tissue model comprises 3 layers: skin, fat, and muscle. A PICO (Smith & Nephew Ltd) 30 x 10-cm single-use NPWT dressing was modeled as a 2-layer structure consisting of a lower adhesive layer and an upper body comprising a spacer layer, a super absorber layer, and a upper moisture vapor transmission layer.^17^ Each FEA model component has a characteristic thickness and a set of mechanical properties that are listed in Table 1. The incision consists of a vertical cut through the skin and 10 mm into the fat. A 50-mm wide horizontal fascial separation was created at the bottom of the incision. Superficial and deep sutures were modeled as 0.5-mm diameter contact points in the skin and fat layers. The mechanical characteristics of tissue have been studied by a number of authors.^19^^,^^20^ While the values cited by different investigators vary widely according to measurement technique and site on the body, the general conclusions are that (i) fat has much lower tensile properties than either skin or muscle and (ii) the behavior of the various components is nonlinear at strains greater than approximately 10% to 20%. The PICO dressing parameters are based on tensile measurements made on the dressings themselves. The components were found to be sufficiently linear within a wide range of strains for a linear model to be used. Table 1 also summarizes the principal material properties included in the model. All are assumed to be isotropic and time independent.

### Biomechanical testing

SynTissue synthetic skin tissue analogue, consisting of 3 layers representing 4-mm skin, 10-mm fat, and 7-mm muscle, was used (Syndaver Labs Inc, Tampa, Fla). Into the synthetic skin, a vertical incision was made through the skin and fat layers down to, but not into, the muscle layer, which was then closed with running stitch using a 2-0 gauge nondissolvable nylon suture (Dafilon™; Aesculap Inc, Center Valley, Pa) 5 mm apart. A horizontal-bed tensile-test machine (Mecmesin Ltd, Sussex, United Kingdom) was used to measure the force required to separate the incision as a function of the magnitude of the negative pressure. Metal plates were attached to the surface of the synthetic skin 10 mm either side of the incision site. These were clamped to the platen of the tensile test machine. By moving the platen at a rate of 2.5 mm/min up to a maximum displacement of 10 mm, the sutured incision site was put under tension and the mechanical response of the tissue was measured (in newton) (see Fig 4). A PICO 10 x 30-cm single-use NPWT dressing was applied to the closed incision. To precisely control the required levels of negative pressure (PICO is designed to operate at −80 mm Hg), a vacuum pump, a needle valve restriction, an accumulator chamber, and a digital pressure gauge were joined to the dressing.

## RESULTS

### FEA computer model

**Figure 2:**
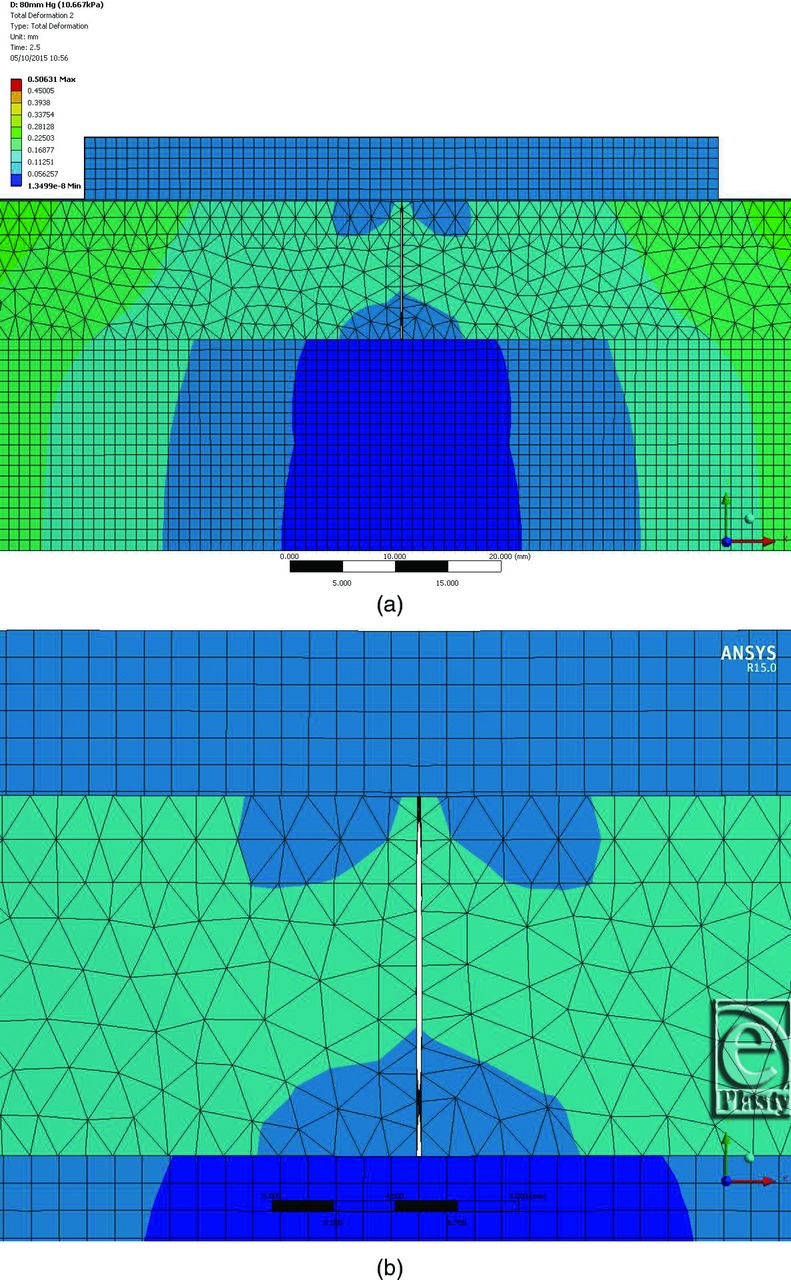

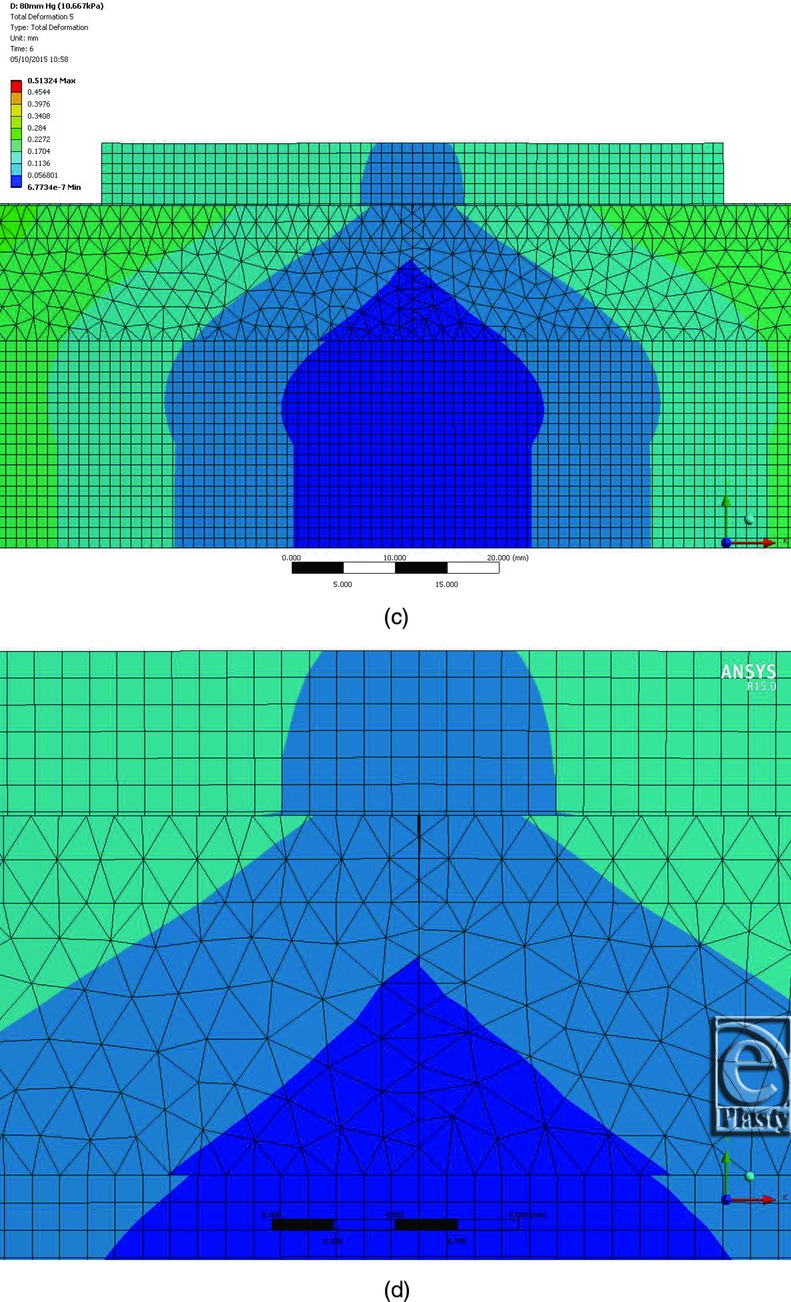
Three-dimensional finite element analysis computer model of skin incision: application of sutures and −80 mm Hg NPWT. (a) Incision model from Figure 1 with sutures applied. Pattern of forces around skin incision in millimeters of tissue deformation from its starting position due to the inherent lateral tensions in the tissue when the incision is made (blue is small; red is larger). The plane of the section is across the incision. (b) Close-up from (a). (c) Incision with NPWT dressing applied (−80 mm Hg). (d) Close-up from (c). NPWT indicates negative pressure wound therapy.

**Figure 3:**
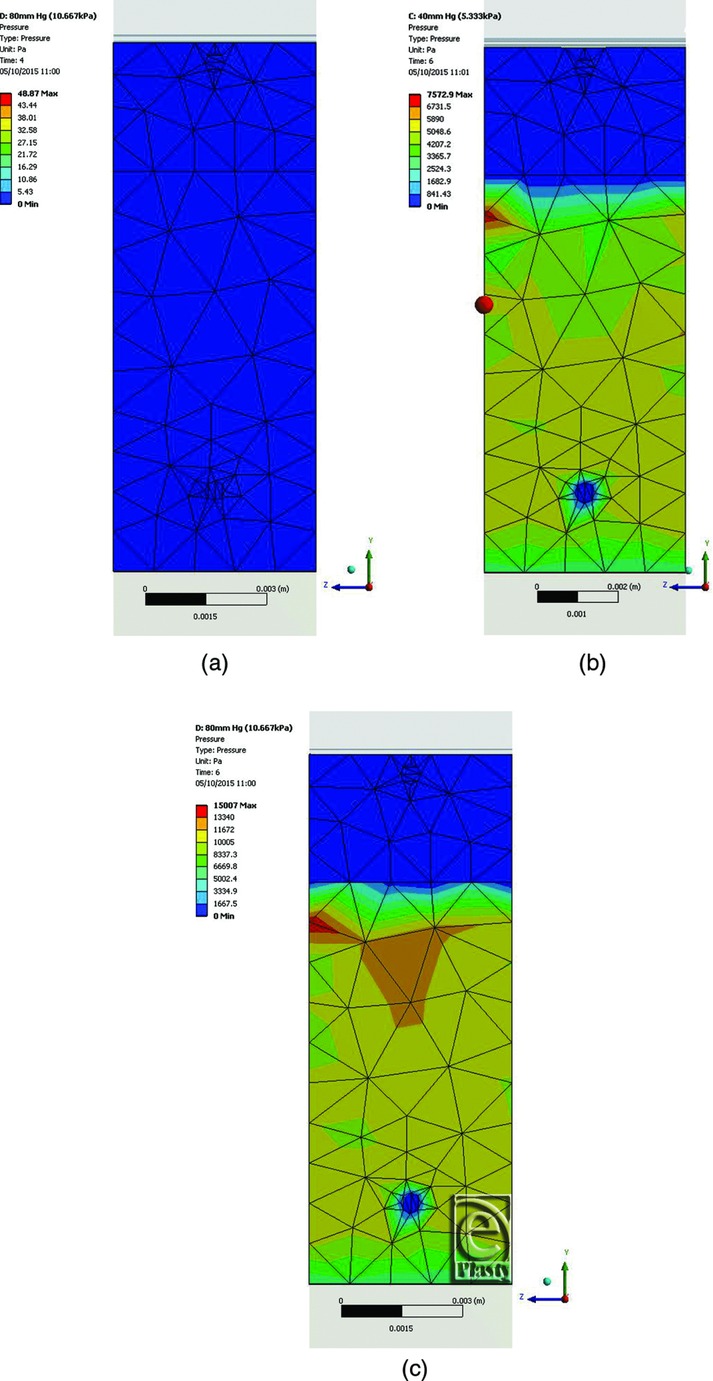
Contact pressure between opposing faces of the vertical incision. (a) The pressures are indicated by color coding (blue is low compression; red is high compression). The plane of the section is through the incision looking directly at one face of the cut tissue: No negative pressure (0.0 kPa). (b) −40 mm Hg (5.3 kPa) negative pressure; average contact pressure 5.2 kPa; maximum contact pressure 7.6 kPa. (c) −80 mm Hg (10.7 kPa) negative pressure; average contact pressure 10.4 kPa; maximum contact pressure 15.0 kPa.

To provide an insight into the mechanisms associated with NPWT and the factors affecting its clinical effects on closed incisions, a 3-dimensional FEA computer model of incision geometry was created. The model enables exploration of the effects that the negative pressure within the dressing has on the incision shape, the contact between opposing faces of the incision, and the stress distribution in the different layers. For simplicity and ease of calculation, a thin slice through the center of an incisional wound was modeled. Given the long aspect ratio of a closed incision, end effects were ignored. Figure 1*a* shows the components of the FEA model, and the color-coded images Figures 1*b* and 1*c* show how the tissue gapes open when the incision is made because of the inherent lateral tensions in the tissue. The plane of the section is across the incision. The deeper blue areas show minimal tissue movement, and the red areas show the maximum deformation (the scale is in millimeters). The wound has a gape of approximately 1.2 mm, given that the tissue on each side has deformed about 0.6 mm. While the PICO dressing is visible in Figure 1, it was “turned off,” that is, it was not mathematically included in the simulation at this point and therefore did not have an effect on the manner in which the tissue gaped following the incision. Figures 2*a* and 2*b* show that the application of sutures tends to pull the incision closed, reducing the magnitude of tissue deformation. The sutures were modeled as 0.5-mm diameter contact points in the skin and fat layers, which restrain the tissue. The color coding in Figures 2*a* and 2*b* can be compared with that in Figures 1*b* and 1*c*; this shows that applying sutures to the incision reduces the gape such that the red areas indicating large displacements disappear. Figures 2*c* and 2*d* then show the effects of applying the PICO dressing and −80 mm Hg of negative pressure to the sutured incision. It is immediately clear from the color coding (darker blue area) that there is a more uniform reduction of lateral tension and the 2 sides of the incision are brought closer together than with sutures alone. The level of negative pressure was altered between −40 mm Hg (not shown) and −80 mm Hg. Within the fat layer, closure is complete at very low negative pressures (a few mm Hg) due to the low elastic strength of the fat layer, which acts like an incompressible quasi fluid. While more gaping occurs within the skin layer than the fat layer due to its inherently stiffer properties, closure is almost complete at negative pressures greater than −40 mm Hg. To investigate the action of negative pressure at the interface between the 2 sides of the incision, Figure 3 shows the contact pressures (in Pa) acting normal to the faces of the incision. The plane of the section is along the incision, looking directly at one face of the cut tissue. Figure 3*a* shows the situation where there is no application of negative pressure, only the sutures. The deep blue coloration indicates little, if any, closing force on the tissue. Figures 3*b* and 3*c* show how the application of −40 and −80 mm Hg negative pressure exerts a compressive closing force on the incision faces. This force will bring the cut tissue edges closer together and resist lateral tension forces across the wound that may prevent or delay healing. The application of negative pressure also offloads the tension that is applied locally to the skin by the sutures. Without application of negative pressure, the force on an individual suture due to natural tension is 1.31 N. The simulation shows that negative pressure of −40 mm Hg reduces this to 0.56 N (43%) and a pressure of −80 mm Hg reduces the force on the suture to 0.4 N (31%). Application of lateral forces to the tissue through the sutures can cause damage, which might predispose to infection. It is clear that the simple FEA model provides evidence that application of negative pressure with a canister-less single-use NPWT system can apply lateral forces to a closed incision and reduce the tension on the sutures in a way similar to that demonstrated for foam-based single-use NPWT systems.^14^

### Biomechanical testing

**Figure 4:**
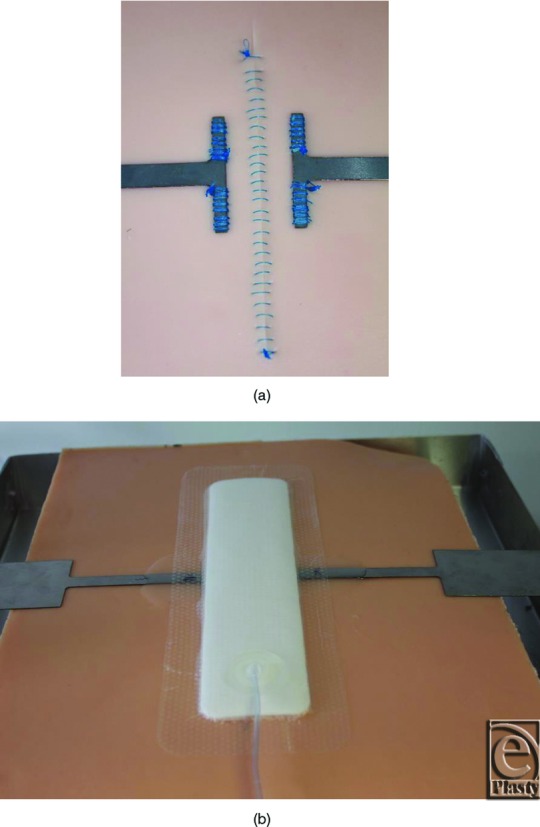
Experimental setup with Syndaver tissue model. (a) Syndaver tissue model with incision. The incision was closed with running stitch using a 2-0 nondissolvable suture 5 mm apart. (b) PICO dressing applied to the top of the Syndaver tissue model.

**Figure 5:**
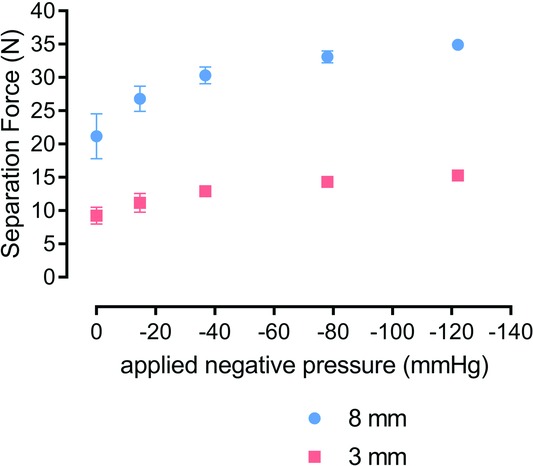
Biomechanical profile with Syndaver tissue model—force at 3 and 8mm extension. Forces required to generate a 3 and 8mm deformation in the Syndaver tissue model at different levels of negative pressure (mm Hg) applied within the PICO single-use dressing. Mean force in newton measured at 0 mm Hg (n = 6); −14.7 mm Hg (n = 5); −36.8 mm Hg (n = 4); 78 mm Hg (n = 4); −122.0 mm Hg (n=3). Errors bars = standard deviation.

**Figure 6:**
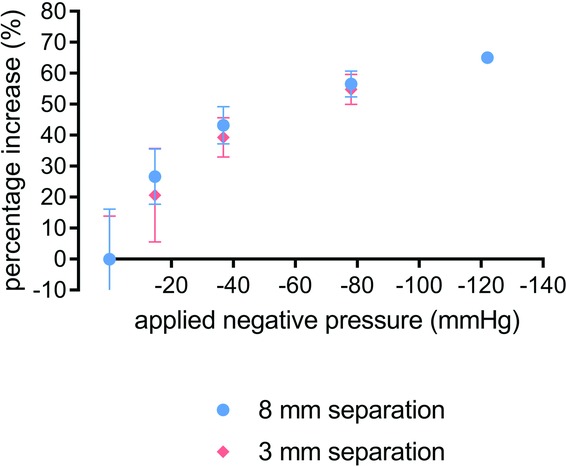
Biomechanical profile with Syndaver tissue model—percent increase in force required for extension of specified length between zero and specified negative pressure. Mean percent increase in force at both 3 mm (red) and 8 mm (blue) measured at 0 mm Hg (n = 6); −14.7 mm Hg (n = 5); −36.8 mm Hg (n = 4); −78 mm Hg (n = 4); −122.0 mm Hg (n = 3). Errors bars = standard deviation.

To confirm the FEA model and to further investigate the effects of NPWT on lateral forces on the closed incision, an in vitro model was set up to allow measurement of the physical force required to separate the sutured incision by applying opposing lateral tension (see Fig 4). The method used a proprietary tissue analogue used for training and teaching surgical techniques, and a 3-layer synthetic skin tissue plate was selected as a test material because it is designed to have mechanical properties similar to human tissue (3 layers: 4mm “skin” layer; 10mm “fat” layer; and 7mm “muscle” layer). Into this synthetic tissue, a vertical incision was made through the skin and fat layers down to, but not into, the muscle layer, which was then closed with running stitch sutures. Figure 5 shows the relationship between the forces required to effect a 3 and 8mm separation of the top edges of the incision in the synthetic tissue model at different levels of NPWT applied to the multilayer PICO dressing. As expected, the force required to generate a 3mm deformation is less than that required to make a greater deformation of 8 mm, but in each case, extra force is required in the presence of NPWT. It appears that most of the effect of the negative pressure to reduce the force required to cause deformation in the model has already been realized at around −40 mm Hg. For example, for an 8-mm deformation, the force required at −40 mm Hg is mean = 30.5 N (n = 5), and there was little more effect at −80 mm Hg (mean = 33.0 N; n = 5) and only slightly more at −120 mm Hg (mean = 35.0 N; n = 5). It is noteworthy that the adhesive multilayered PICO dressing was able to resist lateral tension to a degree, even in the absence of negative pressure. These relationships can be demonstrated further by replotting these results to show the percent increased force required to achieve a given level of displacement at different negative pressures. Figure 6 shows that at pressures of −80 mm Hg, 55% more force was required to get either small (3 mm) or large (8 mm) deformations in the tissue compared with the situation where no NPWT dressing was active. There was a diminishing return at greater negative pressures as the force required for deformation is 65% extra at −120 mm Hg. The effect is consistent at both the 3 and 8mm displacements.

## DISCUSSION

Canister-less single-use NPWT devices represent a new kind of technology with the ability to deliver negative pressure to either an open wound or a closed incision.^17^^,^^18^ A number of clinical studies have shown reductions in the level of surgical site complications such as infection or dehiscence when treated with such a system.^8^^,^^12^^,^^21^^,^^22^ Similar evidence is building for single-use NPWT devices based on open-cell foam.^11^^,^^23^^,^^24^ In an earlier study, Wilkes et al^14^ demonstrated, through FEA modeling and a benchtop tissue model, that a single-use NPWT system based on open-cell foam is able to apply a lateral force to a closed surgical incision. This study has now established that a canister-less NPWT system based on a silicone adhesive multilayer dressing also applies a lateral closing force to surgical incisions. The magnitudes of reduction of lateral force observed in the FEA models in the 2 studies are comparable at around 50%, although the foam-based device uses a negative pressure of −125 mm Hg whereas in the canister-less system, NPWT is set at −80 mm Hg. Both studies used a benchtop synthetic multilayered model of skin to take force measurements in the tissue deformed by laterally applied tensions, with and without the application of negative pressure. In the present study, the percent increase in force required to deform the incision at −80 mm Hg was again around 50%, tallying with the FEA modeling, a result that was also observed in the earlier study.^14^ Analyses of the relationship between the levels of negative pressure applied to the canister-less dressing and the increased resistance to lateral deformation show that at −80 mm Hg, much of the achievable effect (55% increase in separation force to cause a given wound opening) has already been realized because measurements at −120 mm Hg, a 50% increase in negative pressure, show only a small 10% increase in the lateral force that would be required to achieve a comparable deformation. Application of negative pressure to a closed incision undoubtedly operates through multiple mechanisms of action. It is unclear whether an overall optimal level of negative pressure exists, since other parameters, such as the avoidance of ischemia through positive pressure^25^ or damage to healthy skin, are also important considerations.^26^^,^^27^ In clinical studies of other devices designed to reduce only lateral tension across closed incisions, improvements in scar appearance have been reported following prolonged application of detensioning mechanical devices for up to 13 weeks following abdominoplasty.^15^^,^^16^ It will be interesting to discover whether a canister-less single-use NPWT system can also lead to a reduction in scarring through removal of lateral tension and at the same time manage exudate and reduce the level of surgical site complications.

## Figures and Tables

**Table 1:** Finite element analysis computer model: Material properties

	Thickness	Model	Parameters	Source
**Skin**	3.2 mm	Ogden Hyperelastic	μ_1_ = 110 kPa; α_1_ = 9; D_1_ = 0	Shergold and Fleck^19^
**Fat**	20 mm	Ogden Hyperelastic	μ_1_ = 0.4 kPa; α_1_ = 23; *D*_1_ = 0	Comley and Fleck^20^
**Muscle**	10 mm	Ogden Hyperelastic	μ_1_ = 180 kPa; α_1_ = 30; *D*_1_ = 0.25	Wilkes et al^14^
**PICO dressing—lower adhesive layer**	0.15 mm	Linear	*E* = 100 kPa; *ν* = 0.45	This study
**PICO dressing—upper layers**	5.9 mm	Linear	*E* = 800 kPa; *ν* = 0.45	This study
